# Antibiotic Resistance in Gram-Negative Bacteria: The Threat from the Pink Corner

**DOI:** 10.3390/ph17091122

**Published:** 2024-08-25

**Authors:** Alfizah Hanafiah, Bruno S. Lopes

**Affiliations:** 1 Department of Medical Microbiology & Immunology, Faculty of Medicine, Universiti Kebangsaan Malaysia, Cheras, Kuala Lumpur 56000, Malaysia; 2 School of Health & Life Sciences, Room M9.12 (Middlesbrough Tower), Southfield Rd., Middlesbrough TS1 3BX, UK; 3 National Horizons Centre, Teesside University, Darlington DL1 1HG, UK

Antibiotic resistance in Gram-negative bacteria is a formidable challenge in modern medicine [1], often referred to as the threat from the “pink corner” due to the pink staining of these bacteria during Gram staining. This Special Issue, “Antibiotic Resistance in Gram-Negative Bacteria: The Threat from the Pink Corner,” delves into this pressing issue through a compilation of eight research articles and two review papers. The collected works provide a multi-faceted exploration of the mechanisms driving resistance and innovative approaches to the treatment of infectious diseases. By shedding light on the resilience of Gram-negative pathogens, this Special Issue aims to advance our understanding and inform strategies to combat the growing threat of antibiotic resistance, ultimately contributing to global public health efforts. In this Special Issue, multiple aspects of the topic are comprehensively addressed, with particular emphasis on the genomic characterization of multidrug resistance in *Klebsiella pneumoniae*, *Acinetobacter baumannii*, and *Escherichia coli*. Additionally, the antibacterial activity of novel compounds and the mechanisms underlying the development of resistance are thoroughly explored.

According to a study by Mahmud et al. [2], the prevalence and distribution of multidrug-resistant (MDR) and carbapenem-resistant *K. pneumoniae* (CRKP) in Bangladeshi hospitals are concerning. Of the 285 isolates, 67 produced ESBL, and 42 of them were also CRKP, which was mostly discovered on hospital floors and in bed pillows. These isolates showed strong resistance to several drugs, and genes *bla*_TEM_, *bla*_SHV_, and *bla*_CTX-M-1_ were frequently identified. Moreover, almost all isolates exhibited the capacity to produce biofilms and important virulence factors, such as *ugeF* and *fimH*. Possible in-hospital transmission is suggested by clonal correlations seen between patient isolates and ambient isolates [3,4]. These results highlight the critical need for improved antibiotic stewardship and infection control measures to slow the spread of these harmful infectious agents. A study by Yasir et al. [5] revealed significant findings regarding the presence and characteristics of carbapenem-resistant *Acinetobacter baumannii* (CRAB) in a hospital environment. The researchers identified 361 bacterial isolates, predominantly Gram-positive, from hospital surfaces and air samples, with a notable presence of pathogenic and opportunistic pathogens, including CRAB. Genomic analysis of the CRAB isolates from the hospital environment and clinical samples showed the presence of multiple antibiotic resistance genes, including *bla*_OXA-23_ and *bla*_OXA-66_, and highlighted the genetic similarity between environmental and clinical isolates. The study emphasizes the critical need for stringent infection control measures and regular surveillance of hospital environments to manage and reduce the spread of multidrug-resistant pathogens. The research by Altayb et al. [6] demonstrated that both clinical and environmental isolates of *K. pneumoniae* and *E. coli* in Khartoum, Sudan, possess numerous antibiotic resistant genes (ARGs), including β-lactamases and aminoglycoside resistance factors. The isolates were shown to carry ARGs on mobile genetic elements (MGEs), such as plasmids and transposons, which contribute to the dissemination of multidrug-resistant bacteria. The study identified various sequence types (STs) and emphasized the genetic similarities between clinical and environmental isolates, suggesting potential transmission within healthcare settings and the wider community. This highlights the critical need for enhanced infection control strategies and surveillance to mitigate the spread of these resistant pathogens. The importance of infection control measures to reduce the spread of multidrug-resistant pathogens has been investigated by these two studies, which demonstrated that the genomic characteristics of resistant genes indicate potential transmission between clinical and environmental isolates [7].

Important research on the antibacterial activity of synthesized N-(4-bromophenyl)furan-2-carboxamide derivatives is presented in the study by Siddiqa et al. [8]. Compound (3) displayed the highest antibacterial efficacy against multidrug-resistant (MDR) pathogens, especially NDM-positive *A. baumannii*, surpassing numerous commercially available antibiotics. The minimum inhibitory concentration (MIC) and minimum bactericidal concentration (MBC) values for compound (3) indicated its significant inhibitory capability. Additionally, computational docking studies, as well as molecular dynamics (MD) simulations, illustrated that compound (3) was not only able to bind tightly but also remain stable within the active site of NDM-1, thus offering hope for developing compounds with antibacterial activities against resistant bacterial strains. A study by Bersani et al. [9] aims to enhance the efficacy of 1,2,4-triazole-3-thione derivatives as inhibitors of NDM-1 and VIM-type metallo-β-lactamases (MBLs). A key finding is the identification of compounds CP 35, CP 56, and CP 57 as potent micromolar inhibitors that significantly increase bacterial susceptibility to meropenem. Molecular docking and X-ray crystallography revealed that structural modifications, such as adding hydrophobic groups to the triazole ring, improved interactions with the MBL active sites. These optimized inhibitors show great potential for restoring the effectiveness of β-lactam antibiotics against resistant Gram-negative bacteria, addressing a critical need in the fight against antibiotic resistance. This research contributes to the important topic of antibiotic resistance by providing new avenues for therapy and expanding our knowledge of how to successfully tackle resistant bacterial infections. Furthermore, bacteriophages and herbal therapy have shown strong inhibitory effects on bacteria that are resistant to several drugs [10], offering alternate ways to deal with this urgent problem.

Understanding the factors that lead to the development of resistance in pathogens is crucial for elucidating the underlying mechanisms. An investigation into the effect of heavy metal contamination on antibiotic by Ahmed et al. [11], which involved 300 clinically isolated ESBL-producing bacteria from a Pakistani tertiary care hospital, shows that heavy metals—more specifically, arsenic—significantly increase the antibiotic resistance in these bacteria. *Pseudomonas aeruginosa*, *Klebsiella* spp., and *Escherichia coli* were among the species found in the isolates. Results showed that while some antibiotics, such as polymyxin-B and colistin, demonstrated great sensitivity in the absence of heavy metals, their efficacy was significantly decreased in the presence of arsenic. The study concludes that antimicrobial resistance (AMR) is significantly increased by heavy metals, highlighting the need for more research to understand exposure-response relationships and the significance of integrated approaches to manage the growing threat of AMR in healthcare settings and the environment. Merino et al. [12] examined the development of resistance in *Escherichia coli* and *Salmonella typhimurium* when exposed to amoxicillin, colistin, and essential oils (AEN and COLIFIT). Key findings reveal that, while resistance to prolonged antibiotic exposure significantly increased in both bacteria, resistance to essential oils did not. Antibiotic exposure led to substantial increases in minimum inhibitory concentrations (MIC values), but phenotypic analysis showed no changes. Genotypic research identified genetic modifications associated with increased efflux activity and enhanced protection against oxidative stress, particularly in cell binding sites, which were linked to antibiotic resistance and, to a lesser extent, essential oil resistance. These results suggest that essential oils could be a safer alternative to antibiotics, potentially preventing antimicrobial resistance in the agrifood chain. Both studies emphasize how important it is to manage antimicrobial resistance through integrated approaches. Merino et al. propose that essential oils could be a safer alternative to antibiotics in preventing the development of resistance, while Ahmed et al. highlight the role of heavy metals like arsenic in increasing resistance.

Various approaches to combating Gram-negative multidrug-resistant bacteria, highlighted in this Special Issue, emphasize the importance of innovative strategies and integrated efforts to address this critical public health challenge.
